# Supplementary material for: Tlr9 deficiency in B cells leads to obesity by promoting inflammation and gut dysbiosis
Source: Nat Commun. 2024 May 18;15:4232. doi: 10.1038/s41467-024-48611-8 (PMC11102548; doi:10.1038/s41467-024-48611-8)
Supplement: Supplementary file 1 — Supplementary Information [file 41467_2024_48611_MOESM1_ESM.pdf]

Tlr9 deficiency in B cells leads to obesity by promoting inflammatory T cells and gut dysbiosis

Pai Wang [1,2*], Xin Yang[2,3*], Luyao Zhang[1,2], Sha Sha[2,4], Juan Huang [2], Jian Peng[2], Jianlei Gu[5], James Alexander Pearson[2,6], Youjia Hu[2], Hongyu Zhao[5], F. Susan Wong[6], Quan Wang[1†], Li Wen[2†]

1. Department of Gastrocolorectal Surgery, General Surgery Center, The First Hospital of Jilin University, Changchun, Jilin, China.

2. Section of Endocrinology, Department of Internal Medicine, School of Medicine, Yale University, New Haven, Connecticut, USA.

3. Department of Food Science and Technology, School of Agriculture and Biology, Shanghai Jiao Tong University, Shanghai 200240, China.

4. Department of Nephrology, The First Affiliated Hospital of Shandong First Medical University, Jinan, Shandong, China.

5. Department of Biostatistics, Yale School of Public Health, New Haven, Connecticut, USA.

6. Division of Infection and Immunity, School of Medicine and Systems Immunity University Research Institute, Cardiff University, Cardiff, UK.

[*] **These authors contributed equally to this work.**

[†]**Correspondence**: Li Wen, S141, TAC 300, Cedar Street, New Haven, CT 06520-8103, USA; *Telephone: +1 203 785 7186; Fax: +1 203 737 5558; Email: li.wen@yale.edu*.

Quan Wang, Department of Gastrocolorectal Surgery, 71 Xinmin street, Chaoyang District, Changchun, Jilin 130021, China; *Email: wquan@jlu.edu.cn.*

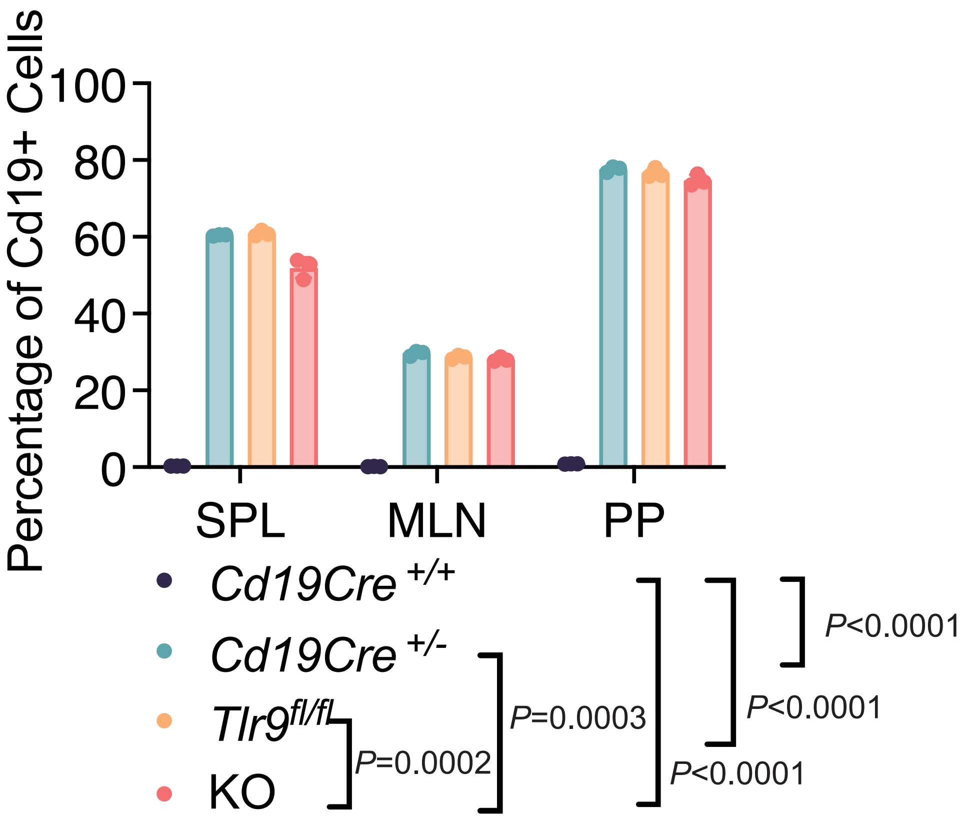

Supplement Figure 1. CD19 expression in lymphoid tissues in different strains of mice. Immune cells from different lymphoid tissues from *CD19Cre*$^{+/+}$, *CD19Cre*$^{+/-}$, *Tlr9*$^{fl/fl}$ and *Tlr9* $^{fl/fl}$*CD19Cre*$^{+/-}$ (KO) 6-8-week-old male mice were prepared and analyzed by flow cytometry after staining with CD19 and Zombie Dye. Flow cytometric plots were gating on CD19^{+} cells. (n=3 mice per group). Data were analyzed by one-way ANOVA. Variations are represented as mean ± SD.

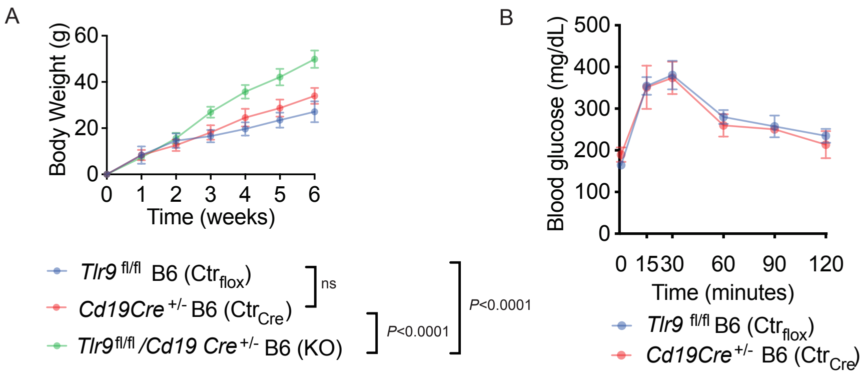

Supplement Figure 2. Metabolic phenotypes of different mice on HFD. (A) Body weight of *Tlr9^fl/fl^* B6 mice (Ctr_{flox}), *Cd19Cre*$^{+/-}$ B6 mice (Ctr_{Cre}) and *Tlr9*$^{fl/fl}$/*Cd19Cre*$^{+/-}$ B6 mice (KO) (Ctr_{flox}: n=4 mice, Ctr_{Cre}: n=7 mice, KO: n=5 mice). **(B)** IPGTT results of the *Tlr9*$^{fl/fl}$ B6 mice (Ctr_{flox}) and *Cd19Cre*$^{+/-}$ B6 mice (Ctr_{Cre}) (Ctr_{flox}: n=6 mice, Ctr_{Cre}: n=5 mice). **(C)** Area under the curve (AUC) of the IPGTT of **(B).** A, B were analyzed by two-way ANOVA and C was analyzed by two-tailed Student's t-test. Variations are represented as mean ± SD.

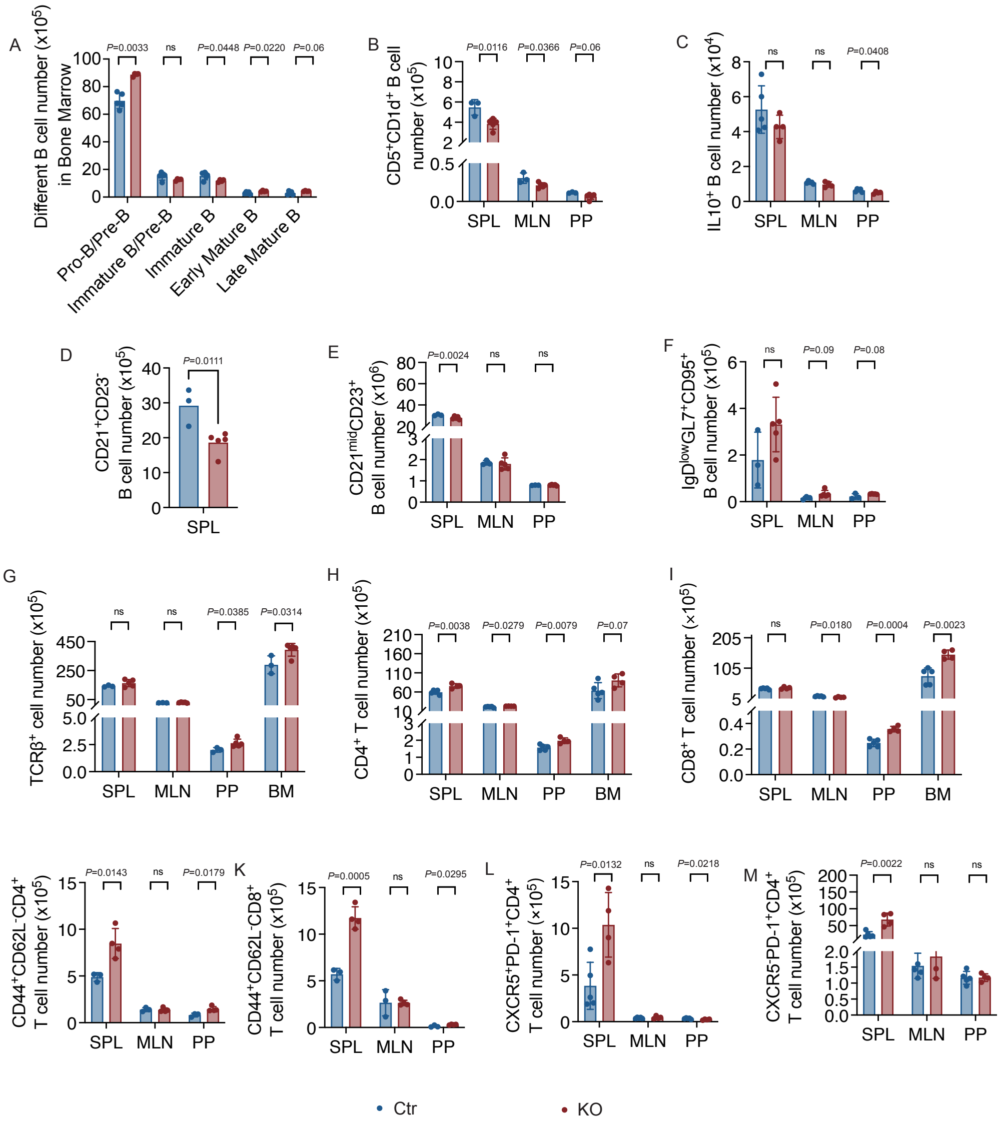

Supplement Figure 3. ***Tlr9*** **deficiency in B cells alters the cell numbers in different immune cell subsets.** Immune cells from different lymphoid tissues (as indicated) from *Tlr9*$^{fl/fl}$*/Cd19Cre*$^{+/-}$ B6 (KO) male mice and *Tlr9*$^{fl/fl}$*/Cd19Cre*$^{-/-}$ B6 control (Ctr) male mice (18-22-week-old) were prepared and analyzed by flow cytometry after staining with different markers and Zombie Dye. All the samples were first gated Zombie Dye negative cells followed by further gating different immune cell subsets. **(A-F)** Immune-phenotype of B cells (gated on B220^{+}) in KO and Ctr mice. **(A)** B cells were first gated on B220^{+} cells. The number of Pro-Pre B cells (CD24^{+},CD43^{-}, IgD^{-}, IgM^{-}), immature-Pre B cells (CD24^{+},CD43^{-}, IgD^{-}, IgM^{mid}), immature B cells (CD24^{+},CD43^{-}, IgD^{-}, IgM^{+}), early mature B cells(CD24^{+},CD43^{-}, IgD^{+}, IgM^{+})

and late mature B cells ($CD24^+$,$CD43^-$, IgD^+, IgM^-), in the bone marrow (KO: n=4 mice, Ctr: n=5 mice). **(B)** The number of $CD5^+CD1d^+$ B cells in SPL, MLN, PP of the KO and the Ctr mice (KO: n=5 mice, Ctr: n=3 mice). **(C)** The number of B cells expressing IL-10 (KO: n=4 mice, Ctr: n=5 mice). **(D)** The number of $CD21^+CD23^-$ marginal zone (MZ) B cells (KO: n=5 mice, Ctr: n=3 mice). **(E)** The number of $CD21^{mid}CD23^+$ follicular (FO) B cells (KO: n=5 mice, Ctr: n=3 mice). **(F)** The number of $IgD^{low}GL7^+CD95^+$ germinal center (GC) B cells (KO: n=5 mice, Ctr: n=3 mice). **(G-M)** Immune-phenotype of T cells (gated on $TCR\beta^+$) in KO mice and Ctr mice by flow cytometric analysis. **(G)** The number of total $TCR\alpha\beta^+$ T cells (KO: n=5 mice, Ctr: n=3 mice). **(H)** The number of $CD4^+$ T cells (KO: n=4 mice, Ctr: n=5 mice). **(I)** The number of $CD8^+$ T cells (KO: n=4 mice, Ctr: n=5 mice). **(J)** The number of $CD44^+CD62L^-$ memory $CD4^+$ T cells. **(K)** The number of $CD44^+CD62L^-$ memory $CD8^+$ T cells (KO: n=4 mice, Ctr: n=3 mice). **(L)** The number of $CXCR5^+$ $PD\text{-}1^+$ follicular T helper (T_{FH}) $CD4^+$ T cells (KO: n=5 mice, Ctr: n=5 mice). **(M)** The number of $CXCR5^-PD\text{-}1^+$ peripheral helper (T_{PH}) $CD4^+$ T cells (KO: n=5 mice, Ctr: n=5 mice). All the experiments were repeated three times with similar results. Data were analyzed using two-tailed Student's *t*-test. The data are presented as mean ± SD. The data presented in the figures are from one of the experiments.

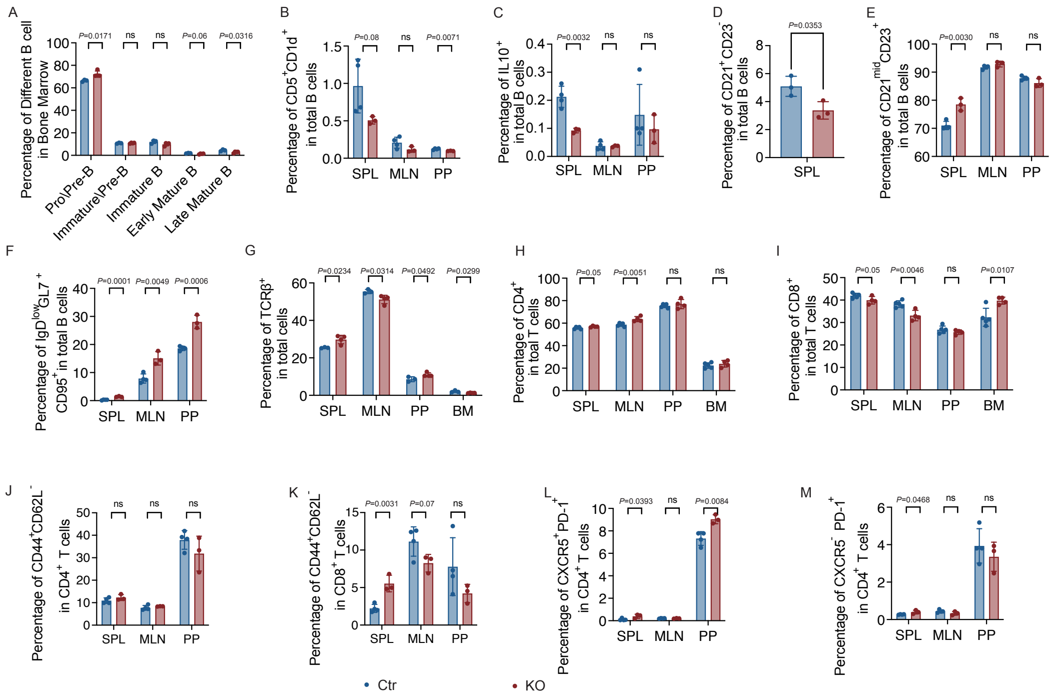

Supplement Figure 4. The immune cell phenotype in mice on normal diet. Mice (6-8 wks old) were sacrificed and immune cells from different lymphoid tissues were isolated and analyzed by flow cytometry after staining with different markers. **(A)** B cell subsets in bone marrow (gated on B220^{+} cells) (KO: n=4 mice, Ctr: n=3 mice). **(B)** Proportion of CD5^{+}CD1d^{+} B cells (KO: n=3 mice, Ctr: n=4 mice). **(C)** Proportion of IL10^{+} expressing B cells (KO: n=3 mice, Ctr: n=4 mice). **(D)** Proportion of CD21^{+}CD23^{-} marginal zone (MZ) B cells (KO: n=3 mice, Ctr: n=3 mice). **(E)** Proportion of CD21^{mid} CD23^{+} follicular (FO) B cells (KO: n=3 mice, Ctr: n=4 mice). **(F)** Proportion of IgD^{low}GL7^{+}CD95^{+} germinal center (GC) B cells (KO: n=3 mice, Ctr: n=4 mice). **(G)** Proportion of total TCRαβ^{+} T cells (KO: n=3 mice, Ctr: n=4 mice). **(H)** Proportion of CD4^{+} T cells (KO: n=5 mice, Ctr: n=5 mice). **(I)** Proportion of CD8^{+} T cells (KO: n=5 mice, Ctr: n=5 mice). **(J)** Proportion of CD44^{+}CD62^{-} memory CD4^{+} T cells (KO: n=3 mice, Ctr: n=4 mice). **(K)** Proportion of CD44^{+}CD62^{-} memory CD8^{+} T cells (KO: n=3 mice, Ctr: n=4 mice). **(L)** frequency of CXCR5^{+}PD-1^{+} follicular T helper (T_{FH}) CD4^{+} T cells (KO: n=3 mice, Ctr: n=4 mice). **(M)** Proportion of CXCR5^{-}PD-1^{+} peripheral helper (T_{PH}) CD4^{+} T cells (KO: n=3 mice, Ctr: n=4 mice). The experiments were repeated twice with similar results and the data from one of the experiments are presented in the figure. Data were analyzed by two-tailed Student's t-test. Variations are represented as mean ± SD.

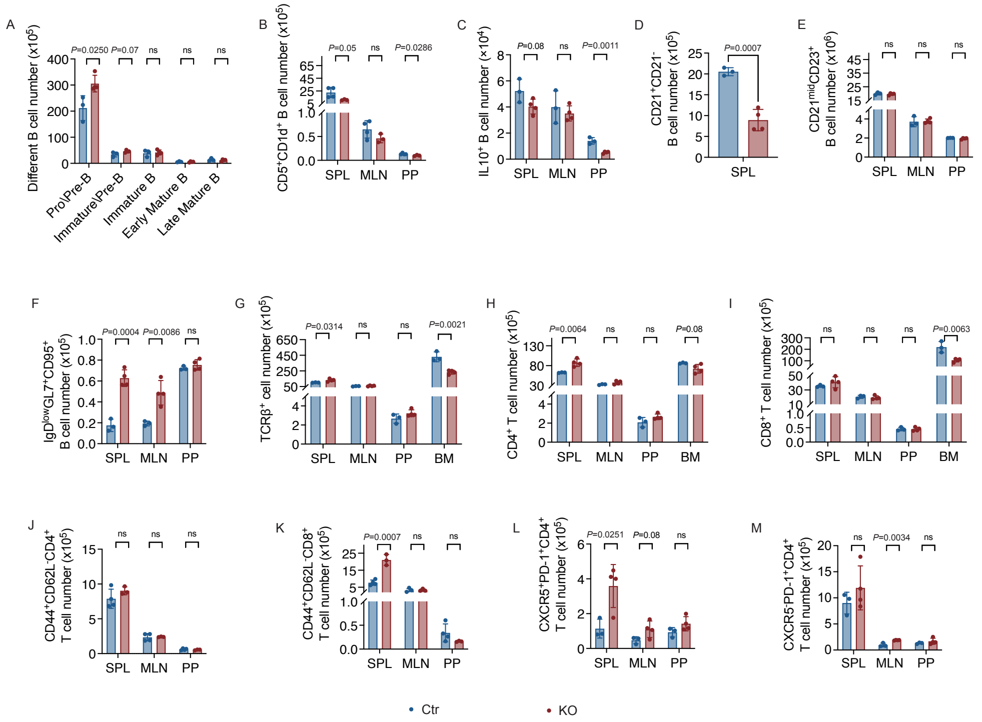

Supplement Figure 5. The number of different immune cell subsets in mice on normal diet. The number of different immune cell subsets from the same mice as in Supplement Figure 4 **(A)** B cell subsets in bone marrow (gated on $B220^+$ cells) (KO: n=4 mice, Ctr: n=3 mice). **(B)** The number of $CD5^+CD1d^+$ B cells (KO: n=3 mice, Ctr: n=4 mice). **(C)** The number of $IL10^+$ expressing B cells (KO: n=4 mice, Ctr: n=3 mice). **(D)** The number of $CD21^+CD23^-$ marginal zone (MZ) B cells (KO: n=4 mice, Ctr: n=3 mice). **(E)** The number of $CD21^{mid}$ $CD23^+$ follicular (FO) B cells (KO: n=4 mice, Ctr: n=3 mice). **(F)** The number of $IgD^{low}GL7^+CD95^+$ germinal center (GC) B cells (KO: n=4 mice, Ctr: n=3 mice). **(G)** The number of total $TCR\alpha\beta^+$ T cells (KO: n=4 mice, Ctr: n=3 mice). **(H)** The number of $CD4^+$ T cells (KO: n=4 mice, Ctr: n=3 mice). **(I)** The number of $CD8^+$ T cells (KO: n=4 mice, Ctr: n=3 mice). **(J)** The number of $CD44^+CD62^-$ memory $CD4^+$ T cells (KO: n=3 mice, Ctr: n=4 mice). **(K)** The number of $CD44^+CD62^-$ memory $CD8^+$ T cells (KO: n=3 mice, Ctr: n=4 mice). **(L)** The number of $CXCR5^+PD\text{-}1^+$ follicular T helper (T_{FH}) $CD4^+$ T cells (KO: n=4 mice, Ctr: n=3 mice). **(M)** The number of $CXCR5^-PD\text{-}1^+$ peripheral helper (T_{PH}) $CD4^+$ T cells (KO: n=4 mice, Ctr: n=3 mice). The experiments were repeated twice with similar results and the data from one of the experiments were presented in the figure. Data were analyzed by two-tailed Student's t-test. Variations are represented as mean ± SD.

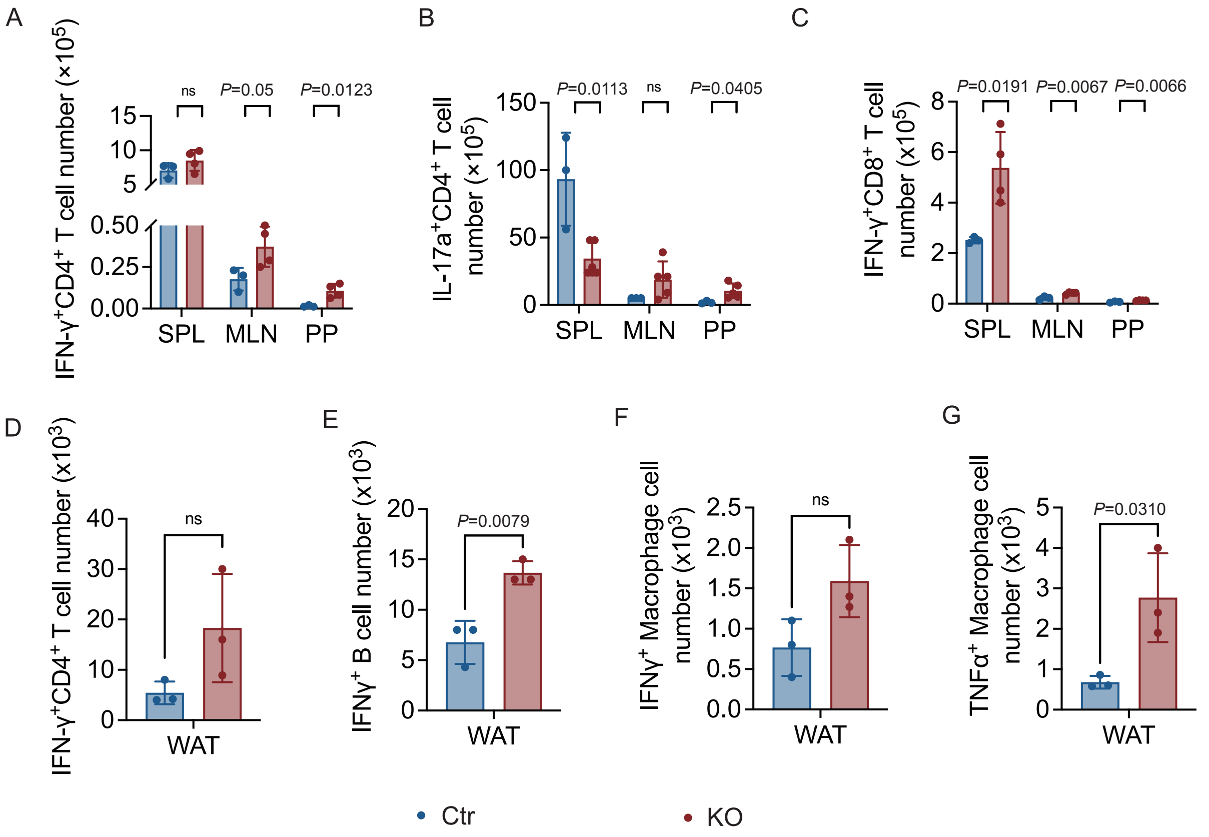

Supplement Figure 6. The number of different immune cells in lymphoid tissues and white adipose tissue (WAT). Mice were sacrificed after 12-16 weeks of HFD and immune cells from different lymphoid tissues and WAT were isolated and analyzed by flow cytometry after staining with various markers. **(A)** The number of IFN-γ expressing $CD4^+$ T cells (KO: n=4 mice, Ctr: n=3 mice). **(B)** The number of IL-17a expressing $CD4^+$ T cells (KO: n=5 mice, Ctr: n=3 mice). **(C)** The number of IFN-γ-expressing $CD8^+$ T cells (KO: n=4 mice, Ctr: n=3 mice). **(D)** The number of IFN-γ-expressing $CD4^+$ T cells (n=3 samples from 6 mice per group). **(E)** The number of IFN-γ-expressing B cells (n=3 samples from 6 mice per group). **(F)** The number of IFN-γ-expressing macrophages (n=3 samples from 6 mice per group). **(G)** The number of TNF-α-expressing macrophages. The experiments were repeated twice with similar results and the data from one of the experiments are presented in the figure. Data were analyzed by two-tailed Student's t-test. Variations are represented as mean ± SD.

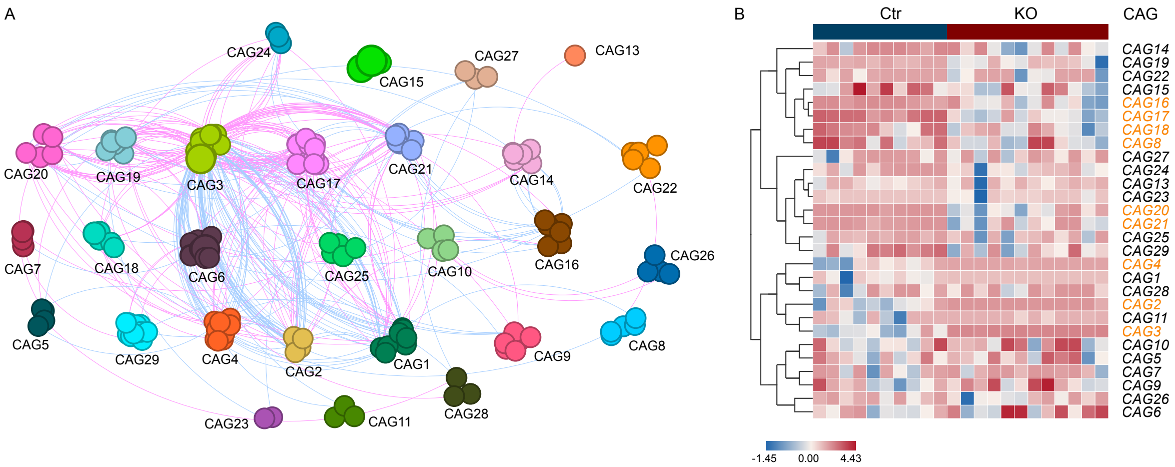

Supplement Figure 7. Microbial interaction network and heatmap of mice on normal diet. **(A)** Microbial interaction network displays the interaction between different Co-abundant groups (CAGs) in mice on normal diet. The size of node represents the mean abundance of amplicon sequencing variant (ASV), being larger size, higher abundance. The lines between the nodes indicate correlation (pink line = negative correlation, blue line = positive correlation), width of the lines corresponding to magnitude of the correlation. **(B)** The heatmap of CAGs in Ctr group and KO group under normal diet (ND). The CAG number marked in bold orange indicate significant differences between the two groups by PERMANOVA.

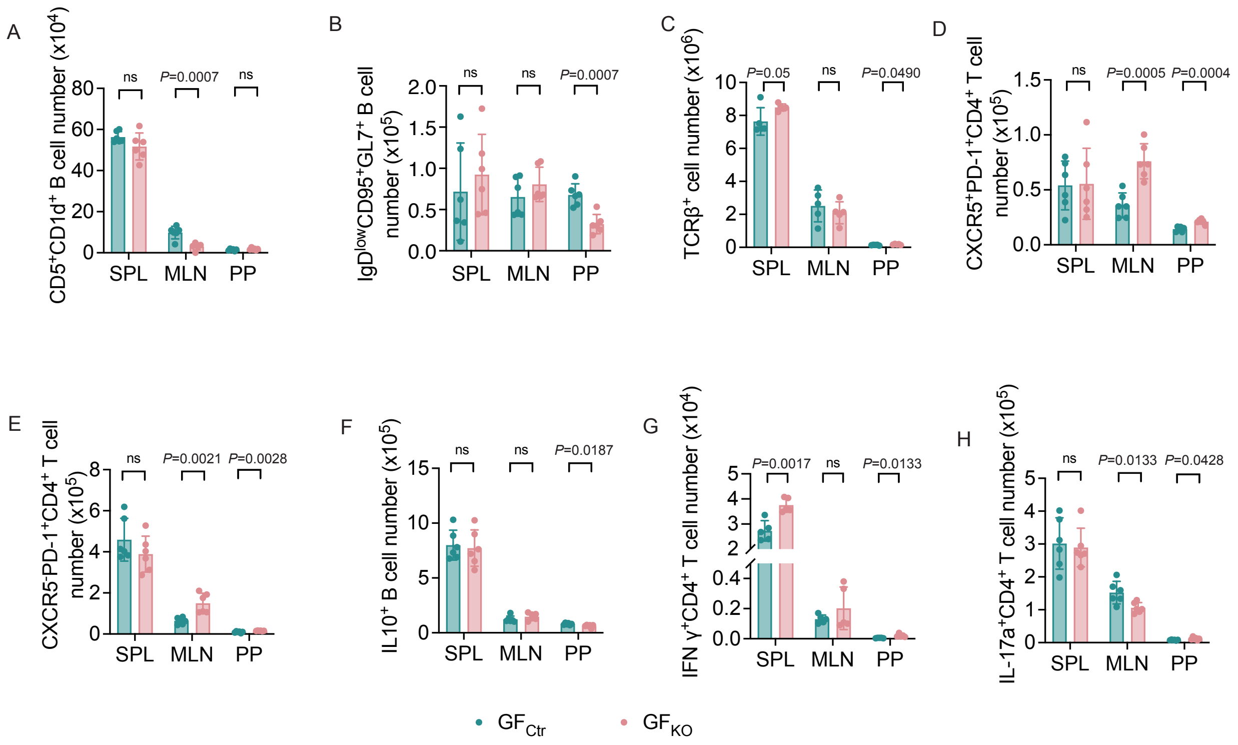

Supplement Figure 8. Immune cell number of ex-GF B6 mice assessed by flow cytometric analysis 14 days after gut microbiota colonization. B cells were gated on B220^{+} and T cells were gated on TCRβ^{+}. **(A)** The number of CD5^{+}CD1d^{+} B cells (gated on B220^{+}) (KO: n=6 mice, Ctr: n=6 mice). **(B)** The number of IgD^{low}GL7^{+}CD95^{+} B cells (KO: n=6 mice, Ctr: n=6 mice). **(C)** The number of total TCRαβ^{+} T cells (KO: n=5 mice, Ctr: n=5 mice). **(D)** The number of CXCR5^{+}PD-1^{+}CD4^{+} T cells (KO: n=6 mice, Ctr: n=6 mice). **(E)** The number of CXCR5^{-}PD-1^{+} CD4^{+} T cells (KO: n=6 mice, Ctr: n=6 mice). **(F)** The number of IL-10-expressing B cells (KO: n=6 mice, Ctr: n=6 mice). **(G)** The number of IFN-γ-expressing CD4^{+} T cells (KO: n=5 mice, Ctr: n=5 mice). **(H)** The number of IL-17a-expressing CD4 T^{+} cells (KO: n=6 mice, Ctr: n=6 mice). The experiments were repeated twice with similar results. The data from one of the experiments are presented in the figure. Data were analyzed by two-tailed Student's t-test. Variations are represented as mean ± SD.

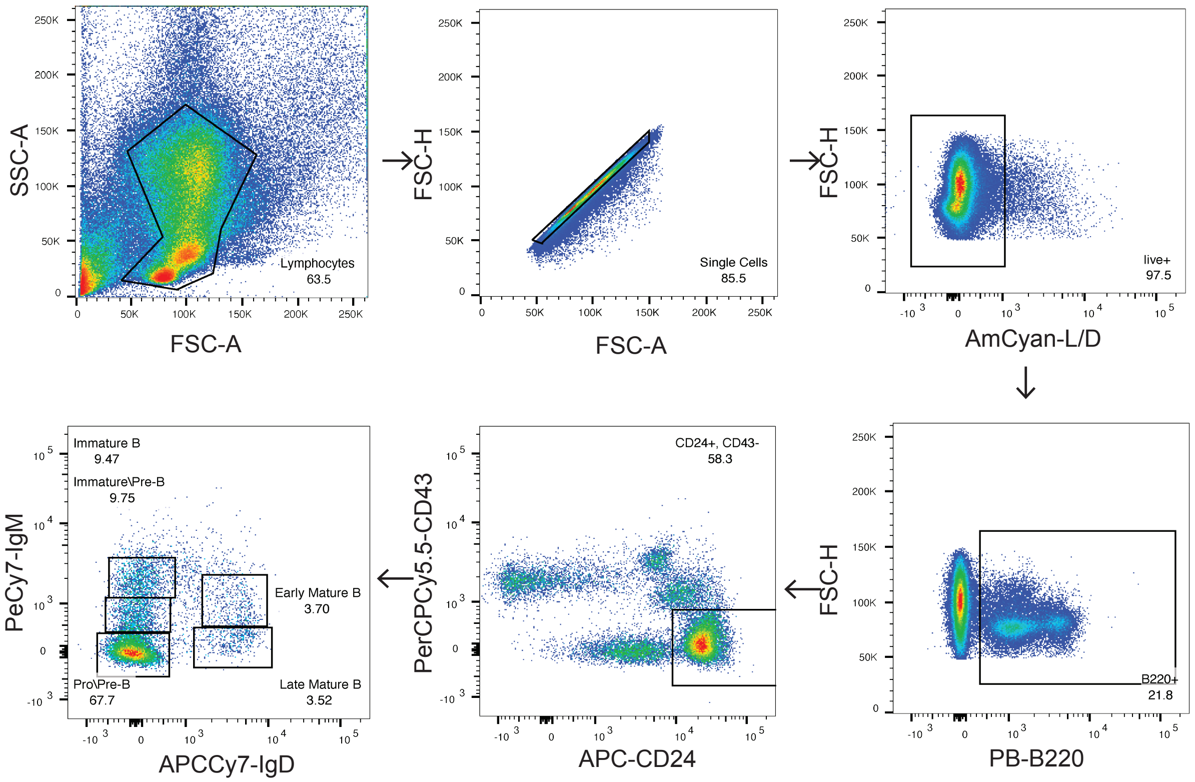

Supplement Figure 9. Gating Strategies of different B cells from bone marrow assessed by flow cytometry. Gating strategies of different B cells from Bone Marrow in Figure 2A, Supplementary Figure 3A, Supplementary Figure 4A and Supplementary Figure 5A.

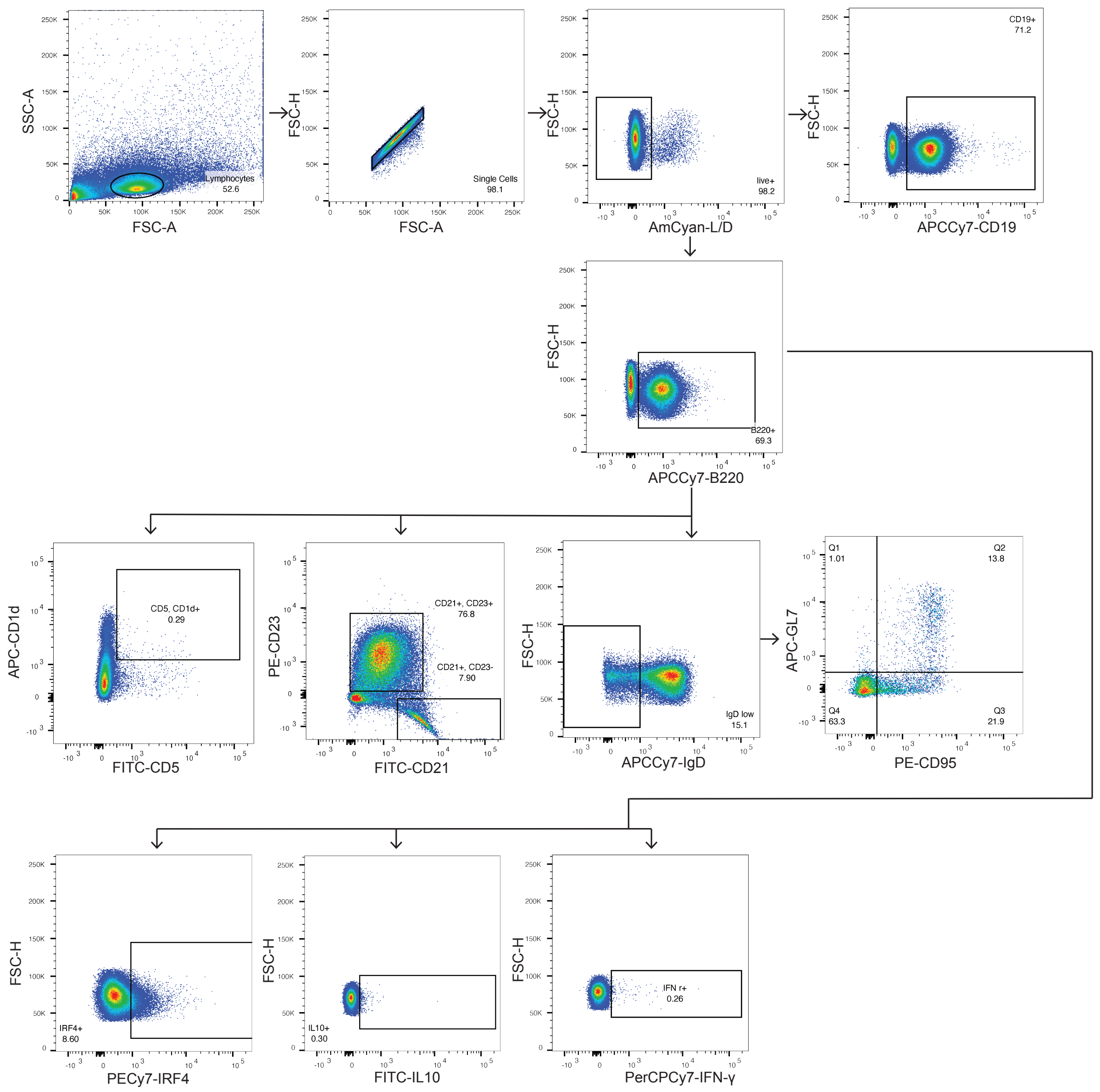

Supplement Figure 10. Gating Strategies of different B cells from bone marrow assessed by flow cytometry. Gating strategies of different B cell subset in Figure 2B-F, Figure 4E, Figure 6D-E, Figure 8H, L, N, O, Q and R, Supplementary Figure 1, Supplementary Figure 3B-F, Supplementary Figure 4B-F and Supplementary Figure 5B-F, Supplementary Figure 6E, Supplementary Figure 8A-B.

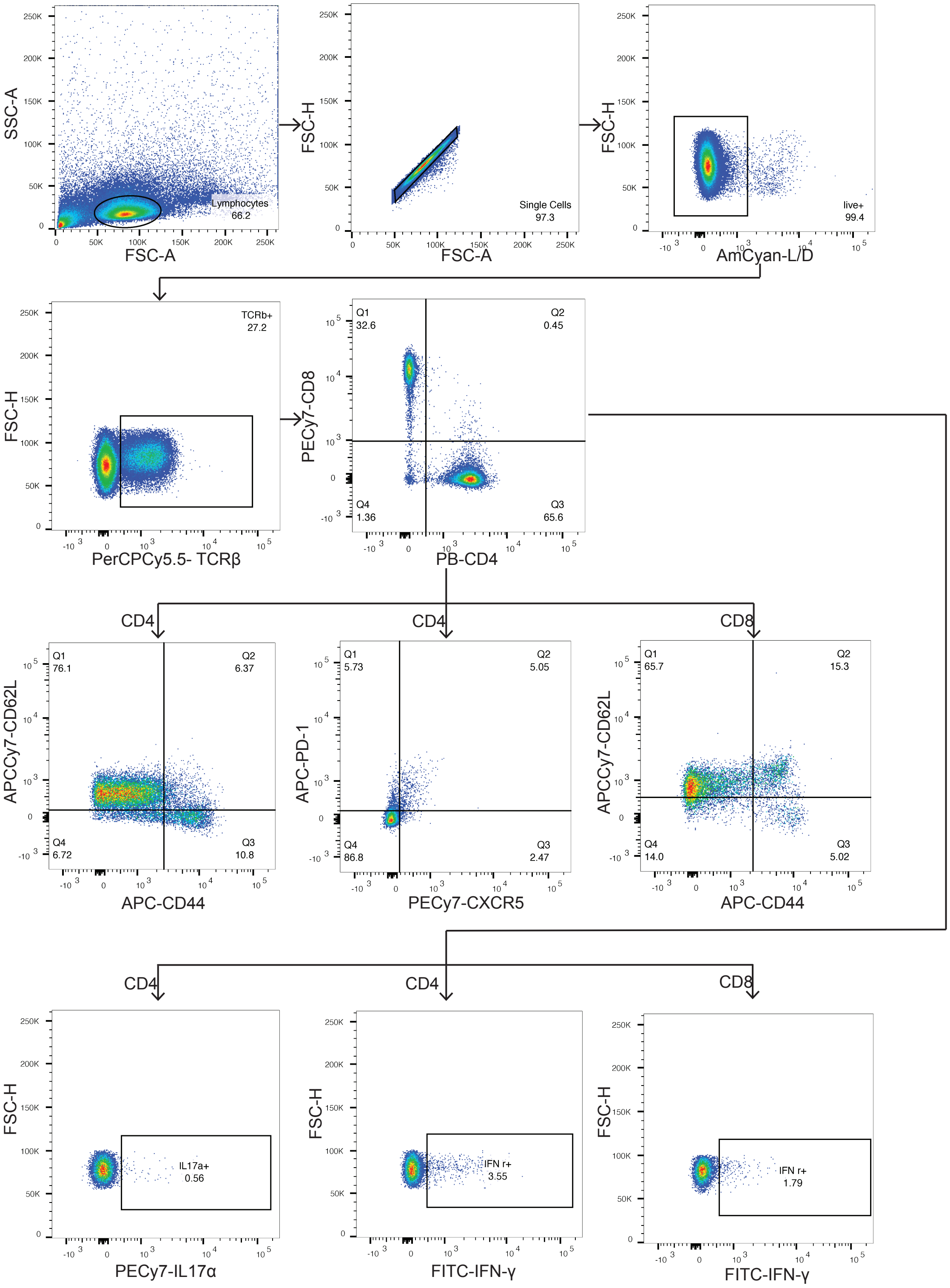

Supplement Figure 11. Gating strategies of different T cells assessed by flow cytometry. Gating strategies of different T cell subsets in Figure 2G-M, Figure 4A-D, Figure 6F-K, Figure 8P and S, Supplementary Figure 3G-M, Supplementary Figure 4G-M, Supplementary Figure 5G-M, Supplementary Figure 6A-D, Supplementary Figure 8C-E and G-H.

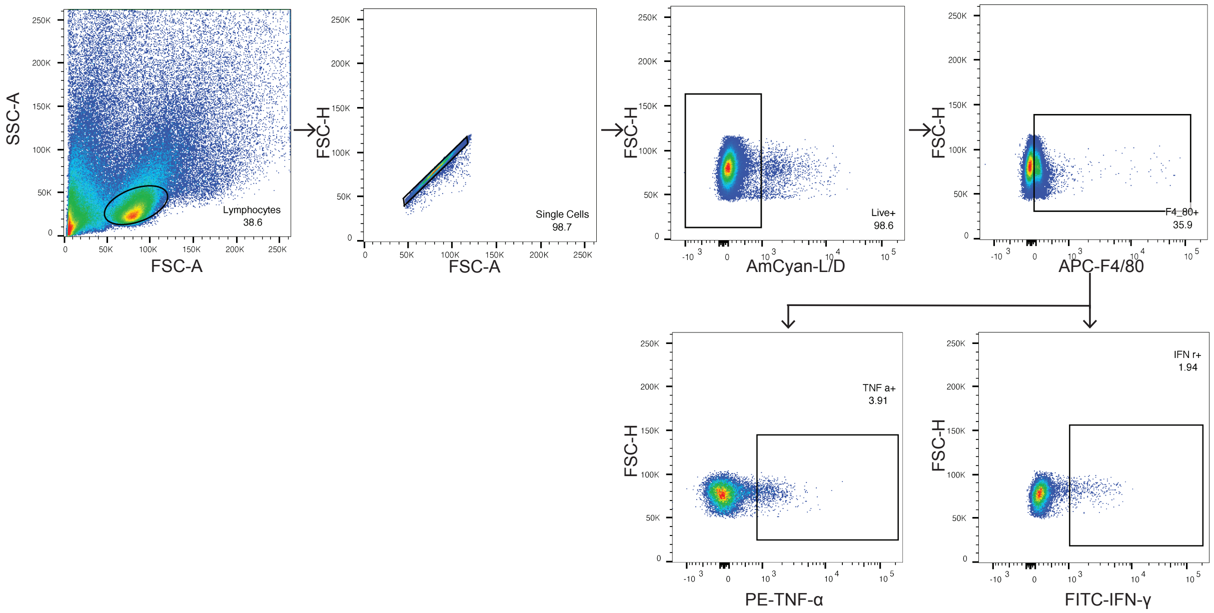

Supplement Figure 12. Gating strategies of Macrophages assessed by flow cytometry. Gating strategies of different expression on macrophages in Figure 4F-G, Supplementary Figure 6F-G.

Supplement Table 1. List of antibodies used in this study.

Target	Type	Catalogue #	Application	Dilution	Source
CD44-APC	Monoclonal	103011	FC	1:1000	Biolegend
TCRβ-PerCPcy5.5	Monoclonal	109227	FC	1:1000	Biolegend
CD8a-PEcy7	Monoclonal	162311	FC	1:1000	Biolegend
CD62L-APCcy7	Monoclonal	104427	FC	1:1000	Biolegend
CD4-PB	Monoclonal	100427	FC	1:1000	Biolegend
CD21-FITC	Monoclonal	123407	FC	1:1000	Biolegend
CD23-PE	Monoclonal	101607	FC	1:1000	Biolegend
CD24-APC	Monoclonal	101813	FC	1:1000	Biolegend
CD43-PerCPcy5.5	Monoclonal	143219	FC	1:1000	Biolegend
IgM-PEcy7	Monoclonal	406513	FC	1:1000	Biolegend
IgD-APCcy7	Monoclonal	405715	FC	1:1000	Biolegend
B220-PB	Monoclonal	103230	FC	1:1000	Biolegend
CD5-FITC	Monoclonal	100605	FC	1:1000	Biolegend
CD1d-APC	Monoclonal	123521	FC	1:1000	Biolegend
B220-APCcy7	Monoclonal	103223	FC	1:1000	Biolegend
PD-1-APC	Monoclonal	109111	FC	1:1000	Biolegend
CXCR5-PEcy7	Monoclonal	145515	FC	1:1000	Biolegend
TCRβ-APCcy7	Monoclonal	109219	FC	1:1000	Biolegend
CD95-PE	Monoclonal	152608	FC	1:1000	Biolegend
GL7-APC	Monoclonal	144617	FC	1:1000	Biolegend
IL-10- FITC	Monoclonal	505005	ICFC	1:1000	Biolegend
IFN-γ-FITC	Monoclonal	505805	ICFC	1:1000	Biolegend
TCRβ-APC	Monoclonal	109211	FC	1:1000	Biolegend
IL-17a-PEcy7	Monoclonal	506939	ICFC	1:1000	Biolegend
CD8a-APCcy7	Monoclonal	100713	FC	1:1000	Biolegend
TNF-a-PE	Monoclonal	506305	ICFC	1:1000	Biolegend
IRF4-PE	Monoclonal	646403	ICFC	1:1000	Biolegend
F4/80-APC	Monoclonal	123115	FC	1:1000	Biolegend

Supplement Table 2. Primer information

Genes	Primers	Sequence
Tlr9	Forward	5′- CCCAGCTTGACAATGAGGTTAT -3′
	Reverse	5′- ACGGGAACTGCTACTACAAGA -3′
Irf4	Forward	5′- TCCGACAGTGGTTGATCGAC-3′
	Reverse	5′- CCTCACGATTGTAGTCCTGCTT-3′
Il-10	Forward	5′- GCATGGCCCAGAAATCAAGG -3′
	Reverse	5′- ACACCTTGGTCTTGGAGCTTATTA-3′
Tnf-α	Forward	5′- CAAATGGCCTCCCTCTCAT-3′
	Reverse	5′- TGGGCTACAGGCTTGTCACT-3′
Ccl2	Forward	5′- TAAAAACCTGGATCGGAACCAAA-3′
	Reverse	5′- GCATTAGCTTCAGATTTACGGGT-3′
Ccl5	Forward	5′- TTTTCAAGGGTCAGTTCCGAC-3′
	Reverse	5′- TTTGCCTACCTCTCCCTCG-3′
Slamf7	Forward	5′- GGCACCTGCGTAATCAATCT-3′
	Reverse	5′- ATGCATGTTAAGGCCTGGTC-3′
Gapdh	Forward	5′- GGGGTCGTTGATGGCAACA-3′
	Reverse	5′- TGTAGACCATGTAGTTGAGGTCA-3′
