# Supplementary material for: Tlr9 deficiency in B cells leads to obesity by promoting inflammation and gut dysbiosis
Source: Nat Commun. 2024 May 18;15:4232. doi: 10.1038/s41467-024-48611-8 (PMC11102548; doi:10.1038/s41467-024-48611-8)
Supplement: Supplementary file 3 — Reporting Summary [file 41467_2024_48611_MOESM3_ESM.pdf]

nature portfolio

Corresponding author(s): Li Wen

Last updated by author(s): Apr 1, 2024

Reporting Summary

Nature Portfolio wishes to improve the reproducibility of the work that we publish. This form provides structure for consistency and transparency in reporting. For further information on Nature Portfolio policies, see our Editorial Policies and the Editorial Policy Checklist.

Statistics

For all statistical analyses, confirm that the following items are present in the figure legend, table legend, main text, or Methods section.

n/a	Confirmed	
☐	☒	The exact sample size (*n*) for each experimental group/condition, given as a discrete number and unit of measurement
☐	☒	A statement on whether measurements were taken from distinct samples or whether the same sample was measured repeatedly
☐	☒	The statistical test(s) used AND whether they are one- or two-sided<br>*Only common tests should be described solely by name; describe more complex techniques in the Methods section.*
☐	☒	A description of all covariates tested
☐	☒	A description of any assumptions or corrections, such as tests of normality and adjustment for multiple comparisons
☐	☒	A full description of the statistical parameters including central tendency (e.g. means) or other basic estimates (e.g. regression coefficient) AND variation (e.g. standard deviation) or associated estimates of uncertainty (e.g. confidence intervals)
☐	☒	For null hypothesis testing, the test statistic (e.g. *F*, *t*, *r*) with confidence intervals, effect sizes, degrees of freedom and *P* value noted<br>*Give P values as exact values whenever suitable.*
☐	☒	For Bayesian analysis, information on the choice of priors and Markov chain Monte Carlo settings
☐	☒	For hierarchical and complex designs, identification of the appropriate level for tests and full reporting of outcomes
☐	☒	Estimates of effect sizes (e.g. Cohen's *d*, Pearson's *r*), indicating how they were calculated

Our web collection on statistics for biologists contains articles on many of the points above.

Software and code

Policy information about availability of computer code

Data collection	All the flow cytometry were collected using Flowjo 10.8.1.
Data analysis	Statistical analysis was performed using GraphPad Prism software v9.0.

For manuscripts utilizing custom algorithms or software that are central to the research but not yet described in published literature, software must be made available to editors and reviewers. We strongly encourage code deposition in a community repository (e.g. GitHub). See the Nature Portfolio guidelines for submitting code & software for further information.

Data

Policy information about availability of data

All manuscripts must include a data availability statement. This statement should provide the following information, where applicable:

- Accession codes, unique identifiers, or web links for publicly available datasets
- A description of any restrictions on data availability
- For clinical datasets or third party data, please ensure that the statement adheres to our policy

The source data is provided with the paper. The 16S rRNA gene sequencing data generated in this study have been deposited in SRA database under accession code PRJNA1093493, https://www.ncbi.nlm.nih.gov/sra/?term=PRJNA1093493. Total B cell RNA sequencing data generated in this study have been deposited in NCBI's Gene Expression Omnibus and are accessible through GEO Series accession number GSE263396 (https://www.ncbi.nlm.nih.gov/geo/query/acc.cgi?acc=GSE263396).

Research involving human participants, their data, or biological material

Policy information about studies with human participants or human data. See also policy information about sex, gender (identity/presentation), and sexual orientation and race, ethnicity and racism.

Reporting on sex and gender	N/A
Reporting on race, ethnicity, or other socially relevant groupings	N/A
Population characteristics	N/A
Recruitment	N/A
Ethics oversight	N/A

Note that full information on the approval of the study protocol must also be provided in the manuscript.

Field-specific reporting

Please select the one below that is the best fit for your research. If you are not sure, read the appropriate sections before making your selection.

☒ Life sciences ☐ Behavioural & social sciences ☐ Ecological, evolutionary & environmental sciences

For a reference copy of the document with all sections, see nature.com/documents/nr-reporting-summary-flat.pdf

Life sciences study design

All studies must disclose on these points even when the disclosure is negative.

Sample size	Sample size chosen was based on 1) G-Power calculation, 2) pilot experiments; 3) availability of all the mouse strains
Data exclusions	no data exclusion
Replication	The the times of replication in each experiment are listed in every figure legend. All the experiments were performed at least twice with similar results.
Randomization	Two types of mice were used in this study - gene knock out mice and control mice. The male mice were used randomly in each group for different experiments.
Blinding	Prior to the experiments, mice were randomly and blinded grouped. Data collection and analysis were semi blinded, as the knockout mice were more obese and the investigators could identify the group without knowing that type of the mice in the experiments.

Reporting for specific materials, systems and methods

We require information from authors about some types of materials, experimental systems and methods used in many studies. Here, indicate whether each material, system or method listed is relevant to your study. If you are not sure if a list item applies to your research, read the appropriate section before selecting a response.

Materials & experimental systems

n/a	Involved in the study	
☐	☒	Antibodies
☒	☐	Eukaryotic cell lines
☒	☐	Palaeontology and archaeology
☐	☒	Animals and other organisms
☒	☐	Clinical data
☒	☐	Dual use research of concern
☒	☐	Plants

Methods

n/a	Involved in the study	
☒	☐	ChIP-seq
☐	☒	Flow cytometry
☒	☐	MRI-based neuroimaging

Antibodies

Antibodies used	The antibodies used in this paper are provided in supplement table 1.<br>Target Type Catalogue # Application Dilution Source

CD44-APC Monoclonal 103011 FC 1:1000 Biolegend
TCRβ-PerCPcy5.5 Monoclonal 109227 FC 1:1000 Biolegend
CD8a-PEcy7 Monoclonal 162311 FC 1:1000 Biolegend
CD62L-APCcy7 Monoclonal 104427 FC 1:1000 Biolegend
CD4-PB Monoclonal 100427 FC 1:1000 Biolegend
CD21-FITC Monoclonal 123407 FC 1:1000 Biolegend
CD23-PE Monoclonal 101607 FC 1:1000 Biolegend
CD24-APC Monoclonal 101813 FC 1:1000 Biolegend
CD43-PerCPcy5.5 Monoclonal 143219 FC 1:1000 Biolegend
IgM-PEcy7 Monoclonal 406513 FC 1:1000 Biolegend
IgD-APCcy7 Monoclonal 405715 FC 1:1000 Biolegend
B220-PB Monoclonal 103230 FC 1:1000 Biolegend
CD5-FITC Monoclonal 100605 FC 1:1000 Biolegend
CD1d-APC Monoclonal 123521 FC 1:1000 Biolegend
B220-APCcy7 Monoclonal 103223 FC 1:1000 Biolegend
PD-1-APC Monoclonal 109111 FC 1:1000 Biolegend
CXCR5-PEcy7 Monoclonal 145515 FC 1:1000 Biolegend
TCRβ-APCcy7 Monoclonal 109219 FC 1:1000 Biolegend
CD95-PE Monoclonal 152608 FC 1:1000 Biolegend
GL7-APC Monoclonal 144617 FC 1:1000 Biolegend
IL-10- FITC Monoclonal 505005 FC 1:1000 Biolegend
IFN-γ-FITC Monoclonal 505805 FC 1:1000 Biolegend
TCRβ-APC Monoclonal 109211 FC 1:1000 Biolegend
IL-17a-PEcy7 Monoclonal 506939 FC 1:1000 Biolegend
CD8a-APCcy7 Monoclonal 100713 FC 1:1000 Biolegend
TNF-a-PE Monoclonal 506305 FC 1:1000 Biolegend
IRF4-PE Monoclonal 646403 FC 1:1000 Biolegend
F4/80-APC Monoclonal 123115 FC 1:1000 Biolegend

Validation

CD44: Each lot of this antibody is quality control tested by immunofluorescent staining with flow cytometric analysis.
Verified Reactivity: Mouse, Human. Application: FC - Quality tested.
Application References
(PubMed link indicates BioLegend citation)
Trowbridge IS, et al. 1982. Immunogenetics 15:299. (ICFC, IP, CMCD)
Katoh S, et al. 1994. J. Immunol. 153:3440. (ELISA)
Budd RC, et al. 1987. J. Immunol. 138:3120. (IP)
Camp RL, et al. 1993. J. Exp. Med. 178:497. (Block)
Weiss JM, et al. 1997. J. Cell Biol. 137:1137. (Block)
Frank NY, et al. 2005. Cancer Res. 65:4320. (IHC) PubMed
Cuff CA, et al. 2001. J. Clin. Invest. 108:1031. (IHC)
Lee JW, et al. 2006. Nature Immunol. 8:181.
Zhang N, et al. 2005. J. Immunol. 174:6967. PubMed
Huabiao C, et al. 2005. J. Immunol. 175:591. PubMed
Gui J, et al. 2007. Int. Immunol. 19:1201. PubMed
Wang XY, et al. 2008. Blood 111:2436. PubMed

TCRβ: Each lot of this antibody is quality control tested by immunofluorescent staining with flow cytometric analysis.
Verified Reactivity: Mouse. Application: FC - Quality tested.
Application References
(PubMed link indicates BioLegend citation)
Gascoigne NJ. 1990. J. Biol. Chem. 265:9296.
Kruisbeek A, et al. 1991. In Current Protocols in Immunology. pp. 3.12.1. (Costim IP)
Davenport C, et al. 1995. J. Immunol. 155:3742. (Costim)
Drobyski W, et al. 1996. Blood 87:5355. (Deplete)
Kummer U, et al. 2001. Immunol. Lett. 75:153. (Deplete)
van der Heyde HC, et al. 1995. J. Immunol. 154:3985. (Deplete)
Tomita K, et al. 1999. Genes Dev. 13:1203. (IHC)
Podd BS, et al. 2006. J. Immunol. 176:6532. (IHC)
Ponomarev ED, et al. 2007. J. Immunol. 178:39. (IHC)
Chappaz S, et al. 2007. Blood doi:10.1182/blood-2007-02-074245. (FC) PubMed
Tsukumo S, et al. 2006. J.Immunol. 177:8365. (FC) PubMed
Grégoire C, et al. 1991. Proc. Natl. Acad. Sci USA 88:8077.

CD8a: Each lot of this antibody is quality control tested by immunofluorescent staining with flow cytometric analysis.
Verified Reactivity: Mouse. Application: FC - Quality tested.
Antigen References
1. Ledbetter JA, et al. 1979. Immunol. Rev. 47:63. (IHC, IP)
2. Hathcock KS. 1991. Current Protocols in Immunology. 3.4.1. (Deplete)
3. Takahashi K, et al. 1992. P. Natl. Acad. Sci. USA 89:5557. (Block, IP)
4. Ledbetter JA, et al. 1981. J. Exp. Med. 153:1503. (Block)
5. Hata H, et al. 2004. J. Clin. Invest. 114:582. (IHC)
6. Fan WY, et al. 2001. Exp. Biol. Med. 226:1045. (IHC)
7. Shih FF, et al. 2006. J. Immunol. 176:3438. (FC)
8. Kamimura D, et al. 2006. J. Immunol. 177:306.
9. Bouwer HGA, et al. 2006. P. Natl. Acad. Sci. USA 103:5102. (FC, Depelete)

10. Kao C, et al. 2005. Int. Immunol. 17:1607. PubMed
11. Ko SY, et al. 2005. J. Immunol. 175:3309. (FC) PubMed
12. Rasmussen JW, et al. 2006. Infect. Immun. 74:6590

CD62L: Each lot of this antibody is quality control tested by immunofluorescent staining with flow cytometric analysis.
Verified Reactivity: Mouse. Application: FC - Quality tested.
Application References
(PubMed link indicates BioLegend citation)
Gallatin WM, et al. 1983. Nature 304:30. (IP, Block)
Siegelman MH, et al. 1990. Cell 61:611. (IP, Block)
Lewinsohn DM, et al. 1987. J. Immunol. 138:4313. (IP, Block)
Iwabuchi K, et al. 1991. Immunobiology 182:161. (CMCD)
Pizcueta P, et al. 1994. Am. J. Pathol. 145:461.
Reichert RA, et al. 1986. J. Immunol. 136:3535. (IHC, FC)
Olver S, et al. 2006. Cancer Res. 66:571.
Fukushima A, et al. 2006. Invest. Ophthalmol. Vis. Sci. 47:657. PubMed
Benson MJ, et al. 2007. J. Exp. Med. doi:10.1084/jem.20070719. (FC) PubMed
Chappaz S, et al. 2007. Blood doi:10.1182/blood-2007-02-074245. (FC) PubMed
Lee JW, et al. 2006. Nature Immunol. 8:181.

CD4: Each lot of this antibody is quality control tested by immunofluorescent staining with flow cytometric analysis.
Verified Reactivity: Mouse. Application: FC - Quality tested.
Application References
(PubMed link indicates BioLegend citation)
Dialynas DP, et al. 1983. J. Immunol. 131:2445. (Block, IP)
Dialynas DP, et al. 1983. Immunol. Rev. 74:29. (IP, Deplete)
Wu L, et al. 1991. J. Exp. Med. 174:1617. (Costim)
Godfrey DI, et al. 1994. J. Immunol. 152:4783. (Block)
Gavett SH, et al. 1994. Am. J. Respir. Cell. Mol. Biol. 10:587. (Deplete)
Schuyler M, et al. 1994. Am. J. Respir. Crit. Care Med. 149:1286. (Deplete)
Ghobrial RR, et al. 1989. Clin. Immunol. Immunopathol. 52:486. (Deplete)
Israelski DM, et al. 1989. J. Immunol. 142:954. (Deplete)
Zheng B, et al. 1996. J. Exp. Med. 184:1083. (IHC)
Frei K, et al. 1997. J. Exp. Med. 185:2177. (IHC)
Felix NJ, et al. 2007. Nat. Immunol. 8:388. (Block)
Radtke AJ, et al. 2020. Proc Natl Acad Sci U S A. 117:33455-65. (SB) PubMed

CD21: Each lot of this antibody is quality control tested by immunofluorescent staining with flow cytometric analysis.
Verified Reactivity: Mouse. Application: FC - Quality tested.
Application References
(PubMed link indicates BioLegend citation)
Boackle S, et al. 2001 Immunity 15:775.
de Andres B, et al. 2012. J. Immunol. 189:2300. PubMed
Chiu YK, et al. 2014. J Immunol. 193:2207. PubMed
Koening PA, et al. 2014. J Biol Chem. 289:34490. PubMed
Radtke AJ, et al. 2020. Proc Natl Acad Sci U S A. 117:33455-65. (SB) PubMed
Radtke AJ, et al. 2022. Nat Protoc. 17:378-401. (SB) PubMed

CD23: Each lot of this antibody is quality control tested by immunofluorescent staining with flow cytometric analysis.
Verified Reactivity: Mouse. Application: FC - Quality tested.
Application References
(PubMed link indicates BioLegend citation)
Waldschmidt TJ, et al. 1988. J. Immunol. 140:2148. (IP)
Rao M, et al. 1987. J. Immunol. 138:1845. (Block)
Oshiba A, et al. 1997. J. Immunol. 159:4056. (Block)
Dasic G, et al. 1999. Eur. J. Immunol. 29:2957. (Block)
Maeda K, et al. 1992. J. Immunol. 148:2340. (IHC)
Craig VJ, et al. 2011. Cancer Res. 71:3616. PubMed
Radtke AJ, et al. 2020. Proc Natl Acad Sci U S A. 117:33455-65. (SB) PubMed
Radtke AJ, et al. 2022. Nat Protoc. 17:378-401. (SB) PubMed

CD24: Each lot of this antibody is quality control tested by immunofluorescent staining with flow cytometric analysis.
Verified Reactivity: Mouse. Application: FC - Quality tested.
Application References
(PubMed link indicates BioLegend citation)
Springer T, et al. 1978. Eur. J. Immunol. 8:539. (WB)
Crowley M, et al. 1989. Cell. Immunol. 118:108. (FA)
Veillette A, et al. 1989. J. Exp. Med. 170:1671. (FA)
Pandelakis A Flavell RA 1999 JEM 189:855 (FC, IHC)
Liu JQ, et al. 2007 J. Immunol. 178:6227. (FC, IF)
Chappaz S, et al. 2007. Blood doi:10.1182/blood-2007-02-074245. (FC) PubMed
Rucci F, et al. 2010. Proc Natl Acad Sci USA. 107:3024. (FC) PubMed
Teague TK, et al. 2010. Int Immunol. 22:387. (FC) PubMed
Gracz AD, et al. 2010. Am J. Physiol Gastrointest Liver Physiol. 298:590. (FC) PubMed
Chen CY, et al. 2008. Endocrinology. 10:1210. (FC, IHC) PubMed
Qui Q, et al. 2010. J. Immunol. 184:1681. (FC) PubMed

CD43: Each lot of this antibody is quality control tested by immunofluorescent staining with flow cytometric analysis.
Verified Reactivity: Mouse. Application: FC - Quality tested.
Application References
(PubMed link indicates BioLegend citation)
Gaspari AA, et al. 1993. J. Invest. Dermatol. 100:247. (FC)
Merzaban JS, et al. 2005. J. Immunol. 174:4051. (FC)
Baecher-Allan CM, et al. 1993. Immunogenetics. 37:183. (WB)

IgM: Each lot of this antibody is quality control tested by immunofluorescent staining with flow cytometric analysis.
Verified Reactivity: Mouse. Application: FC - Quality tested.
Application References
(PubMed link indicates BioLegend citation)
Tertilt C, et al. 2009. Infect. Immun. 77:3044. (ELISA) PubMed

IgD: Each lot of this antibody is quality control tested by immunofluorescent staining with flow cytometric analysis.
Verified Reactivity: Mouse. Application: FC - Quality tested.
Application References
(PubMed link indicates BioLegend citation)
Nitschke L, et al. 1993. P. Natl. Acad. Sci. USA 90:1887. (FC)
Weih D, et al. 2001. J. Immunol. 167:1909. (IHC)
Koni PA, et al. 2001. J. Exp. Med. 193:741. (IHC)
Ahuja A, et al. 2007. J. Immunol. 179:3351. (FC) PubMed
Haynes NM, et al. 2007. J. Immunol. 179:5099. (FC)
Good-Jacobson KL, et al. 2010. Nat. Immunol. 11:535. (FC) PubMed
Tomayko MM, et al. 2010. J. Immunol. 185:7146. PubMed
Park SY, et al. 2013. J. Immunol. 190:1094. PubMed
Rouaud P, et al. 2014. J Exp Med. 211:975. PubMed
Radtke AJ, et al. 2020. Proc Natl Acad Sci U S A. 117:33455-65. (SB) PubMed
Radtke AJ, et al. 2022. Nat Protoc. 17:378-401. (SB) PubMed

B220: Each lot of this antibody is quality control tested by immunofluorescent staining with flow cytometric analysis.
Verified Reactivity: Mouse, Human. Application: FC - Quality tested.
Application References
(PubMed link indicates BioLegend citation)
Coffman RL. 1982. Immunol. Rev. 69:5. (IP)
George A, et al. 1994. J. Immunol. 152:1014. (Activ)
Asensi V, et al. 1989. Immunology 68:204. (Activ)
Domiati-Saad R, et al. 1993. J. Immunol. 151:5936. (Activ)
Hata H, et al. 2004. J. Clin. Invest. 114:582. (IHC)
Monteith CE, et al. 1996. Can. J. Vet. Res. 60:193. (IHC)
Shih FF, et al. 2006. J. Immunol. 176:3438. (FC)
Chang C L-T, et al. 2007. J. Immunol. 178:6984.
Fazilleau N, et al. 2007. Nature Immunol. 8:753.
Lang GL, et al. 2008. Blood 111:2158. PubMed
Charles N, et al. 2010. Nat. Med. 16:701. (FC) PubMed
del Rio ML, et al. 2011. Transpl. Int. 24:501. (FC) PubMed

CD5: Each lot of this antibody is quality control tested by immunofluorescent staining with flow cytometric analysis.
Verified Reactivity: Mouse. Application: FC - Quality tested.
Application References
(PubMed link indicates BioLegend citation)
Ledbetter JA, et al. 1979. Immunol. Rev. 47:63. (IP)
Ledbetter JA, et al. 1980. J. Exp. Med. 152:280. (FC, IHC)
Bourdeau A, et al. 2007. Blood doi:10.1182/blood-2006-08-044370.

CD1d:Each lot of this antibody is quality control tested by immunofluorescent staining with flow cytometric analysis.
Verified Reactivity: Mouse. Application: FC - Quality tested.
Application References
(PubMed link indicates BioLegend citation)
Fischer K, et al. 2004. P. Natl. Acad. Sci. USA 101:10685. (Block)
Brozovic S, et al. 2004. Nat. Med. 10:535.
Brossay L, et al. 1997. J. Immunol.. 159:1216. (Block)
Jiang J, et al. 2012. PLoS One. 7:47487. PubMed

B220: Each lot of this antibody is quality control tested by immunofluorescent staining with flow cytometric analysis.
Verified Reactivity: Mouse, Human. Application: FC - Quality tested.
Application References
(PubMed link indicates BioLegend citation)
Coffman RL. 1982. Immunol. Rev. 69:5. (IP)
George A, et al. 1994. J. Immunol. 152:1014. (Activ)
Asensi V, et al. 1989. Immunology 68:204. (Activ)
Domiati-Saad R, et al. 1993. J. Immunol. 151:5936. (Activ)
Hata H, et al. 2004. J. Clin. Invest. 114:582. (IHC)
Monteith CE, et al. 1996. Can. J. Vet. Res. 60:193. (IHC)
Shih FF, et al. 2006. J. Immunol. 176:3438. (FC)

Chang C L-T, et al. 2007. J. Immunol. 178:6984.
Fazilleau N, et al. 2007. Nature Immunol. 8:753.
Lang GL, et al. 2008. Blood 111:2158. PubMed
Charles N, et al. 2010. Nat. Med. 16:701. (FC) PubMed
del Rio ML, et al. 2011. Transpl. Int. 24:501. (FC) PubMed

PD-1: Each lot of this antibody is quality control tested by immunofluorescent staining with flow cytometric analysis.
Verified Reactivity: Mouse. Application: FC - Quality tested.
Application References
(PubMed link indicates BioLegend citation)
Matsumoto K, et al. 2004. J. Immunol. 172:2530.
Raimondi G, et al. 2006. J. Immunol. 176:2808. (FC) PubMed
King IL, et al. 2009. J. Exp Med 206:1001. (FC) PubMed

CXCR5: Each lot of this antibody is quality control tested by immunofluorescent staining with flow cytometric analysis.
Verified Reactivity: Mouse. Application: FC - Quality tested.
Product Citations
Rydyznski CE et al. 2018. Cell reports. 24(13):3367-3373 . PubMed
Zhang R, et al. 2021. Cell Mol Immunol. 18:1222. PubMed
Manion KP, et al. 2020. PLoS One. 15:e0236664. PubMed
Shan M et al. 2018. Immunity. 49(4):709-724 . PubMed
Boyoglu-Barnum S, et al. 2014. J Virol. 88:10569. PubMed
Cohen CA, et al. 2021. Nat Commun. 12:4678. PubMed

TCRβ: Each lot of this antibody is quality control tested by immunofluorescent staining with flow cytometric analysis.
Verified Reactivity: Mouse. Application: FC - Quality tested.
Application References
(PubMed link indicates BioLegend citation)
Gascoigne NJ. 1990. J. Biol. Chem. 265:9296.
Kruisbeek A, et al. 1991. In Current Protocols in Immunology. pp. 3.12.1. (Costim IP)
Davenport C, et al. 1995. J. Immunol. 155:3742. (Costim)
Drobyski W, et al. 1996. Blood 87:5355. (Deplete)
Kummer U, et al. 2001. Immunol. Lett. 75:153. (Deplete)
van der Heyde HC, et al. 1995. J. Immunol. 154:3985. (Deplete)
Tomita K, et al. 1999. Genes Dev. 13:1203. (IHC)
Podd BS, et al. 2006. J. Immunol. 176:6532. (IHC)
Ponomarev ED, et al. 2007. J. Immunol. 178:39. (IHC)
Chappaz S, et al. 2007. Blood doi:10.1182/blood-2007-02-074245. (FC) PubMed
Tsukumo S, et al. 2006. J.Immunol. 177:8365. (FC) PubMed
Grégoire C, et al. 1991. Proc. Natl. Acad. Sci USA 88:8077.

CD95: Each lot of this antibody is quality control tested by immunofluorescent staining with flow cytometric analysis.
Verified Reactivity: Mouse. Application: FC - Quality tested.
Product Citations
Trittel S, et al. 2019. Sci Rep. 9:16362. PubMed
Zhang R, et al. 2021. Cell Mol Immunol. 18:1222. PubMed
Kim CJ, et al. 2018. Immunity. 49:1034. PubMed
Woodruff MC et al. 2018. Cell reports. 25(2):321-327 . PubMed
Pobezinskaya EL et al. 2019. Front Immunol. 1.079861111 . PubMed
Waide ML, et al. 2020. Cell Rep. 33:108503. PubMed
Wang J, et al. 2018. Front Immunol. 9:371. PubMed
Zhang YN, et al. 2020. EBioMedicine. 56:102819. PubMed
Beloor J et al. 2018. Cell host & microbe. 23(4):549-556 . PubMed
Kang X, et al. 2022. J Immunol Res. 2022:8118577. PubMed
Mandal RK, et al. 2021. Cell Reports. 35(6):109094. PubMed
Lazarian G, et al. 2021. Cancer Cell. 39(3):380-393.e8. PubMed

GL7: Each lot of this antibody is quality control tested by immunofluorescent staining with flow cytometric analysis.
Verified Reactivity: Mouse, Human. Application: FC - Quality tested.
Application References
(PubMed link indicates BioLegend citation)
Laszlo G, et al. 1993. J. Immunol. 150:5252. (FC, IP)
Hartgring SA, et al. 2012. Arthritis Res. Ther. 14:R137. (FC)
Taylor JJ, et al. 2012. J. Exp. Med. 209:597. (FC, IHC)
Balogh A, et al. 2010. Immunol. Lett. 130:89. (IHC)
Kimura N, et al. 2007. J. Biol. Chem. 282:32200. (ELISA, FC)

IL-10: Each lot of this antibody is quality control tested by intracellular immunofluorescent staining with flow cytometric analysis.
Verified Reactivity: Mouse. Application: ICFC - Quality tested.
Application References
(PubMed link indicates BioLegend citation)
Simkin G, et al. 2000. J. Immunol. 164:2457.
Kitagaki K, et al. 2002. Clin. Diagn. Lab Immunol. 9:1260.
Khanna A, et al. 2000. J. Immunol. 164:1346.
Sander B, et al. 1993. J. Immunol. Methods 166:201.
Litton M, et al. 1994. J. Immunol. Methods 175:47.

Andersson U, et al. 1999. Detection and qunatification of gene expression. New York:Springer-Verlag.
Finkelman F, et al. 2003. Curr. Prot. Immunol. John Wiley & Sons New York. Unit 6.28.
Wang W, et al. 2004. FASEB J. 18:1043.
Brummel R and Lenert P. 2005. J. Immunol. 174:2429.
Lawson BR, et al. 2007. J. Immunol. 178:5366.
Xu G, et al. 2007. J. Immunol. 179:5358. PubMed
Brummel R, et al. 2005. J. Immunol.174:2429. PubMed

IFN-γ: Each lot of this antibody is quality control tested by intracellular immunofluorescent staining with flow cytometric analysis.
Verified Reactivity: Mouse. Application: ICFC - Quality tested.
Application References
(PubMed link indicates BioLegend citation)
Abrams J, et al. 1992. Immunol. Rev. 127:5. (ELISA, Neut)
Sander B, et al. 1993. J. Immunol. Meth. 166:201. (ELISA, Neut)
Abrams J, et al. 1995. Curr. Prot. Immunol. John Wiley and Sons, New York. Unit 6.20. (ELISA, Neut)
Yang X, et al. 1993. J. Immunoassay 14:129. (ELISA)
Klinman D, et al. 1994. Curr. Prot. Immunol. John Wiley and Sons, New York. Unit 6.19. (ELISPOT)
Sander B, et al. 1991. Immunol. Rev. 119:65. (IHC)
Ferrick D, et al. 1995. Nature 373:255. (FC)
Ko SY, et al. 2005. J. Immunol. 175:3309. (FC) PubMed
Peterson KE, et al. 2000. J. Virol. 74:5363. (Neut)
DeKrey GK, et al. 1998. Infect. Immun. 66:827. (Neut)
Dzhagalov I, et al. 2007. J. Immunol. 178:2113. (ELISA)
Lawson BR, et al. 2007. J. Immunol. 178:5366. (FC)

TCRβ: Each lot of this antibody is quality control tested by immunofluorescent staining with flow cytometric analysis.
Verified Reactivity: Mouse. Application: FC - Quality tested.
Application References
(PubMed link indicates BioLegend citation)
Gascoigne NJ. 1990. J. Biol. Chem. 265:9296.
Kruisbeek A, et al. 1991. In Current Protocols in Immunology. pp. 3.12.1. (Costim IP)
Davenport C, et al. 1995. J. Immunol. 155:3742. (Costim)
Drobyski W, et al. 1996. Blood 87:5355. (Deplete)
Kummer U, et al. 2001. Immunol. Lett. 75:153. (Deplete)
van der Heyde HC, et al. 1995. J. Immunol. 154:3985. (Deplete)
Tomita K, et al. 1999. Genes Dev. 13:1203. (IHC)
Podd BS, et al. 2006. J. Immunol. 176:6532. (IHC)
Ponomarev ED, et al. 2007. J. Immunol. 178:39. (IHC)
Chappaz S, et al. 2007. Blood doi:10.1182/blood-2007-02-074245. (FC) PubMed
Tsukumo S, et al. 2006. J.Immunol. 177:8365. (FC) PubMed
Grégoire C, et al. 1991. Proc. Natl. Acad. Sci USA 88:8077.

IL17a: Each lot of this antibody is quality control tested by intracellular immunofluorescent staining with flow cytometric analysis.
Verified Reactivity: Mouse. Application: ICFC - Quality tested.
Application References
(PubMed link indicates BioLegend citation)
Kennedy J, et al. 1996. J. Interferon Cytokine Res. 16:611.
Schubert D, et al. 2004. J. Immunol. 172:4503. (ICFC)
Infante-Duarte C, et al. 2000. J. Immunol. 165:6107. (ICFC, ELISA Capture)
Harrington LE, et al. 2005. Nature Immunol. doi:10.1038/ni1254. (ICFC, ELISA Capture)
Nekrasova T, et al. 2005. J. Immunol. 175:2734. (ELISPOT Capture)
Yen D, et al. 2006. J. Clin. Invest. 116:1310. (Neut)
Ehirchiou D, et al. 2007. J. Exp. Med. 204:1519. (ICFC)
Kang SG, et al. 2007. J. Immunol. 179:3724. (ICFC)
Smith E, et al. 2008. J. Immunol. 181:1357. (Neut) PubMed
Neufert C, et al. 2007. Eur. J. Immunol. 37:1809. PubMed
Wang C, et al. 2009. Mucosal Immunol 2:173. (ICFC) PubMed
Cui Y, et al. 2009. Invest. Ophth. Vis. Sci. 50:5811. (ICFC) PubMed

CD8a: Each lot of this antibody is quality control tested by immunofluorescent staining with flow cytometric analysis.
Verified Reactivity: Mouse. Application: FC - Quality tested.
Application References
(PubMed link indicates BioLegend citation)
Ledbetter JA, et al. 1979. Immunol. Rev. 47:63. (IHC, IP)
Hathcock KS. 1991. Current Protocols in Immunology. 3.4.1. (Deplete)
Takahashi K, et al. 1992. P. Natl. Acad. Sci. USA 89:5557. (Block, IP)
Ledbetter JA, et al. 1981. J. Exp. Med. 153:1503. (Block)
Hata H, et al. 2004. J. Clin. Invest. 114:582. (IHC)
Fan WY, et al. 2001. Exp. Biol. Med. 226:1045. (IHC)
Shih FF, et al. 2006. J. Immunol. 176:3438. (FC)
Kamimura D, et al. 2006. J. Immunol. 177:306.
Bouwer HGA, et al. 2006. P. Natl. Acad. Sci. USA 103:5102. (FC, Deplete)
Kao C, et al. 2005. Int. Immunol. 17:1607. PubMed
Ko SY, et al. 2005. J. Immunol. 175:3309. (FC) PubMed
Rasmussen JW, et al. 2006. Infect. Immun. 74:6590. PubMed

TNF-a: Each lot of this antibody is quality control tested by intracellular immunofluorescent staining with flow cytometric analysis.
Verified Reactivity: Mouse. Application: ICFC - Quality tested.
Application References
(PubMed link indicates BioLegend citation)
Abrams J, et al. 1992. Immunol. Rev. 127:5. (Neut)
Abrams J, et al. 1995. Curr. Prot. Immunol. John Wiley and Sons, New York. Unit 6.20
Mo X, et al. 1995. J. Virol. 69:1288.
Sarawar S, et al. 1994. J. Immunol. 153:1246.
Via C, et al. 2001. J. Immunol. 167:6821. (Neut)
Infante-Duarte C, et al. 2000 J. Immunol. 165:6107. (FC)
Jacobs M, et al. 2000. Immunology 100:494. (IHC)
Marinova-Mutachieva L, et al. 1997. Clin. Exp. Immunol. 107:507. (IHC)
Williams RO, et al. 2000. J. Immunol. 165:7240. (IHC)
Scanga CA, et al. 1999. Infect. Immun. 67:4531. (Neut)
Akilov OE, et al. 2007. J. Leukoc. Biol. 2007;10.1189/jlb.0706439. (FC)
Lawson BR, et al. 2007. J. Immunol. 178:5366. (FC)

IRF4: Each lot of this antibody is quality control tested by intracellular immunofluorescent staining with flow cytometric analysis.
Verified Reactivity: Mouse. Application: ICFC - Quality tested.
Application References
(PubMed link indicates BioLegend citation)
Zheng Y, et al. 2009. Nature 458:351
Yin SY, et al. 2011. Exp Cell Res. 317:2210. PubMed
Radtke AJ, et al. 2020. Proc Natl Acad Sci USA. 117:33455-33465. (SB) PubMed
Radtke AJ, et al. 2022. Nat Protoc. 17:378-401. (SB) PubMed

F4/80: Each lot of this antibody is quality control tested by immunofluorescent staining with flow cytometric analysis.
Verified Reactivity: Mouse. Application: FC - Quality tested.
Application References
(PubMed link indicates BioLegend citation)
Schaller E, et al. 2002. Mol. Cell. Biol. 22:8035. (IHC)
Stevceva L, et al. 2001. BMC Clin Pathol. 1:3. (IHC)
Kobayashi M, et al.2008. J. Leukoc. Biol. 83:1354. PubMed
Poeckel D, et al. 2009. J. Biol Chem. 284:21077. PubMed
Glass AM, et al. 2013. J. Immunol. 190:4830. PubMed
Koehm S, et al. 2007. J. Allergy Clin. Immunol. 120:570. (IHC)
Rankin AL, et al. 2010. J. Immunol. 184:1526. (IHC)
Sasi SP, et al. 2014. J Biol Chem. 289:14178. PubMed
Thakus VS, et al. 2014. Toxicol Lett. 230:322. PubMed
Watson NB, et al. 2015. J Immunol. 194:2796. PubMed
Hirakawa H, et al. 2015. PLoS One. 10:119360. PubMed
Radtke AJ, et al. 2020. Proc Natl Acad Sci U S A. 117:33455-65. (SB) PubMed

Animals and other research organisms

Policy information about studies involving animals; ARRIVE guidelines recommended for reporting animal research, and Sex and Gender in Research

Laboratory animals

Tlr9fl/fl C57BL/6 breeder mice were kindly provided by Dr. Mark Shlomchik (University of Pittsburgh). Cd19Cre+/+C57BL/6 breeders were purchased from The Jackson Laboratory (RRID: IMSR_JAX:006785). Tlr9fl/fl and Cd19Cre+/+ B6 mice were bred to generate Tlr9fl/fl/Cd19Cre+/-, which were then intercrossed to obtain B cell–specific Tlr9-deficient (Tlr9fl/fl/Cd19Cre+/-) and control (Tlr9fl/fl/Cd19Cre-/-) B6 mice.
C57BL/6 mice were originally purchased from the Jax Lab (#000664).
Germ-free (GF) C57BL/6 breeders were kindly provided by Dr. Richard Flavell (Yale University) and bred in the Yale gnotobiotic mouse facility.
Rag1-/- B6 mice were originally from the Jax Lab (#002216) and Rag1-/- GF C57BL/6 breeders were kindly provided by Dr. Noah Palm (Yale University) and maintained in the Yale gnotobiotic mouse facility.
Rag1-/- B6 mice used in this study were conventionalized from Rag1-/- GF C57BL/6 mice and kept in SPF environment.
Body weight observation/ immune cell phenotype/ gut microbiota investigations in the experiments of high fat diet fed Tlr9fl/fl/Cd19Cre+/- (KO) and Tlr9fl/fl/Cd19Cre-/- (Ctr) mice were done when the mice were 18-22-week old. The immune phenotype of the KO and Ctr mice used in normal food diet experiments were 6-8-week old. The germ free mice were gavaged when they were 4-week-old, and observed for 2 weeks or 4 weeks. The Rag-/- B6 mice was transfered cells when 4 weeks old and observed for 14-18 week (18-22-week old). The Rag1-/- GF C57BL/6 mice were gavaged and transfered immune cells when 4 weeks old and observed 4 weeks (8-week-old).

Wild animals

No wild animals were used in the study

Reporting on sex

It is well established by scientists in the filed that male mice are a better sex for high fat diet induced obesity (HFDIO) and most of published studies in HFDIO used male mice. Our study also used male mice.

Field-collected samples

No field collected samples were used in the study

Ethics oversight | All the experimental procedures were in accordance with federal regulation and all the protocols used for animal experimentation were approved by Yale Animal Care and Use Committee of Yale University.

Note that full information on the approval of the study protocol must also be provided in the manuscript.

Plants

Seed stocks: N/A

Novel plant genotypes: N/A

Authentication: N/A

Flow Cytometry

Plots

Confirm that:

- ☐ The axis labels state the marker and fluorochrome used (e.g. CD4-FITC).
- ☒ The axis scales are clearly visible. Include numbers along axes only for bottom left plot of group (a 'group' is an analysis of identical markers).
- ☒ All plots are contour plots with outliers or pseudocolor plots.
- ☒ A numerical value for number of cells or percentage (with statistics) is provided.

Methodology

Sample preparation: Bone marrow (BM), spleen (SPL), mesenteric lymph nodes (MLN) and Peyer's patches (PP), were dissected and collected into test tubes containing 3-5ml sterile PBS. All the tissues were homogenized by gently grinding the tissue with the frosted end of glass slides followed by filtering through a nylon filter (150µm) to remove debris. Red blood cells in splenocytes were lysed by hypotonic shock.

Instrument: BD LSR II flow cytometer.

Software: Flowjo

Cell population abundance: No sorting by flow cytometry

Gating strategy: The cells were first gated on live and single cells, followed by gating out the cells that were stained with control antibodies. The cells were than further gated on the populations of interest.

☒ Tick this box to confirm that a figure exemplifying the gating strategy is provided in the Supplementary Information.
