# Supplementary material for: Tlr9 deficiency in B cells leads to obesity by promoting inflammation and gut dysbiosis
Source: Nat Commun. 2024 May 18;15:4232. doi: 10.1038/s41467-024-48611-8 (PMC11102548; doi:10.1038/s41467-024-48611-8)
Supplement: Supplementary file 4 — Description of additional supplementary files [file 41467_2024_48611_MOESM4_ESM.docx]

**Description of additional supplementary files**

**Supplementary Data 1:**

The complete data on the relative abundance and taxonomic status of ASVs in the CAGs matrix of the HFD.

**Supplementary Data 2:**

The complete data on the relative abundance and taxonomic status of ASVs in the CAGs matrix of the ND.
